# Pancytopenia as an initial manifestation of prostate cancer: a case report

**DOI:** 10.1186/s13256-021-02843-0

**Published:** 2021-05-19

**Authors:** Marcos Antonio Custódio Neto da Silva, Vitor Pimentel Rodrigues Manhães, Luadir Gasparotto Júnior, Daniela Miti Lemos Tsukumo, Cristina Alba Lalli

**Affiliations:** Faculty of Medical Science, Medical Residency Program in Internal Medicine, Clinical Hospital from State University of Campinas, Rua Tessália Vieira de Camargo, 126. Cidade Universitária Zeferino Vaz. CEP, 13083-887, Campinas, Sao Paulo Brasil

**Keywords:** Prostate cancer, Bone marrow invasion, Metastasis

## Abstract

**Background:**

Prostate adenocarcinoma is the most frequent cancer type among men, followed by skin cancer. Patients with prostate cancer usually present lower urinary tract symptoms due to tumor involvement. Bone marrow invasion is associated with prostate cancer metastasis and is common if blastic lesions in bones are present but is very rare without a large bone involvement and uncommon as initial presentation.

**Case presentation:**

We present a case of an 86-year-old Caucasian man with bone marrow invasion of prostate cancer without urological or bone-related symptoms and without prostate nodules. His findings were dyspnea, fatigue, and tachycardia. We detail the complete investigation of the case until we found the accurate diagnosis. The patient started treatment, but he had no response and so the oncology team started palliative care.

**Conclusion:**

Bone marrow invasion as an initial manifestation of prostate cancer is not common, especially if no prostatic lesions are found. This report is important to provide additional information about prostate cancer management.

## Background

Cancer is a world health problem. Cancer is the second leading cause of deaths globally according to the World Health Organization (WHO). In 2013, the International Agency on Cancer Research (IARC) estimated that there would be 18.1 million new cancer cases and 9.6 million cancer deaths in 2018 [1].

Prostate cancer is the most common cancer among men, followed by skin cancer, and the second most frequent cause of cancer death among men [1]. In 2018, 1,276,106 cases occurred and 358,989 deaths due to prostate cancer were registered. Prostate adenocarcinoma is the most frequent cancer in men in Brazil. The National Institute of Cancer (INCA) estimated that there were 68,220 prostate cancer cases in 2018–2019 [2].

The main risk factors for development of prostate cancer are age > 50 years [3] or age > 45 years with a family history of prostate cancer [4] and being African-American [5]. Furthermore, men with prostate-specific antigen (PSA) levels > 1 ng/mL at 40 years and > 2 ng/mL at 60 years are also at increased risk of metastasis and death [6, 7].

Prostate cancer is initially suspected by digital rectum examination and/or PSA levels. Definitive diagnosis depends on histopathological verification of adenocarcinoma in prostate biopsy [8].

Metastatic prostate cancer is usually common in bones, lung, and liver and has a 5-year survival rate of 29.3% [9]. Androgen deprivation therapy (ADT) has been traditionally used for the treatment of newly diagnosed metastatic prostate cancer [10]. More recently, some trials have incorporated early chemotherapy with taxanes along with ADT. Zhu et al. [11] showed a synergistic effect of taxanes and ADT, blocking microtubule activity mediated by androgen receptors.

Here, we present the case of an 86-year-old man with an atypical presentation of prostate cancer with biopsy-proven bone marrow invasion without huge involvement of bones and without visible prostate nodules.

## Case report

An 86-year-old Caucasian man presented to the emergency department with a chief complaint of worsening dyspnea.

One month prior to admission, he started with progressive dyspnea on minimal effort, fatigue, and palpitations. He had undergone examinations and consultation with a cardiologist, who identified arrhythmia and started medication (metoprolol 25 mg). Because of the continuation of symptoms, after 1 week, a geriatrician performed general examinations, like a hemogram, PSA, urea, creatinine, and albumin. The examinations evidenced hemoglobin 12.0 g/dL, total PSA was 130 ng/mL, and free PSA > 20 ng/mL. The patient stated that the last PSA done 2 years ago was normal. Because of this, he was referred to a urologist, who requested kidney and urinary tract ultrasound (US) and bone scintigraphy. US showed normal prostate size (10 cm^3^) and no nodularity. Scintigraphy showed probable bone metastases in the blades and spinous process of L4 vertebra and right iliac crest.

Because of persistent symptoms, he underwent new tests 1 month later; these showed that hemoglobin was 6.2 g/dL, platelets 51,000/µL, leukocytes 3950/µL, total PSA 84 ng/mL, and free PSA > 20.0 ng/mL. Because of these examinations, he was referred to an oncologist, who prescribed degarelix (240 mg—first dose and 80 mg once per week for maintenance) as palliative treatment for metastatic prostate cancer and indicated transfusion.

The patient attended the emergency department with frank dyspnea 15 days after degarelix initiation. Tests were performed that showed pancytopenia, and blood transfusion was indicated (2 units of red blood cells). The patient was hospitalized in the Medical Clinic Nursery for investigation of the condition.

The patient was evaluated by the hematology team, who performed a peripheral blood smear that showed marked anisocytosis at the expense of macrocytosis and elliptocytes, thrombocytopenia, and hypogranulation of neutrophils with segmentation failure. Examinations requested for pancytopenia investigation revealed the following: normal vitamin B_12_ (482 pmol/L), normal folic acid (7.6 ng/mL), reticulocytosis (5.2%), and increased lactate dehydrogenase (LDH) (350 IU/L)

**Fig. 1:**
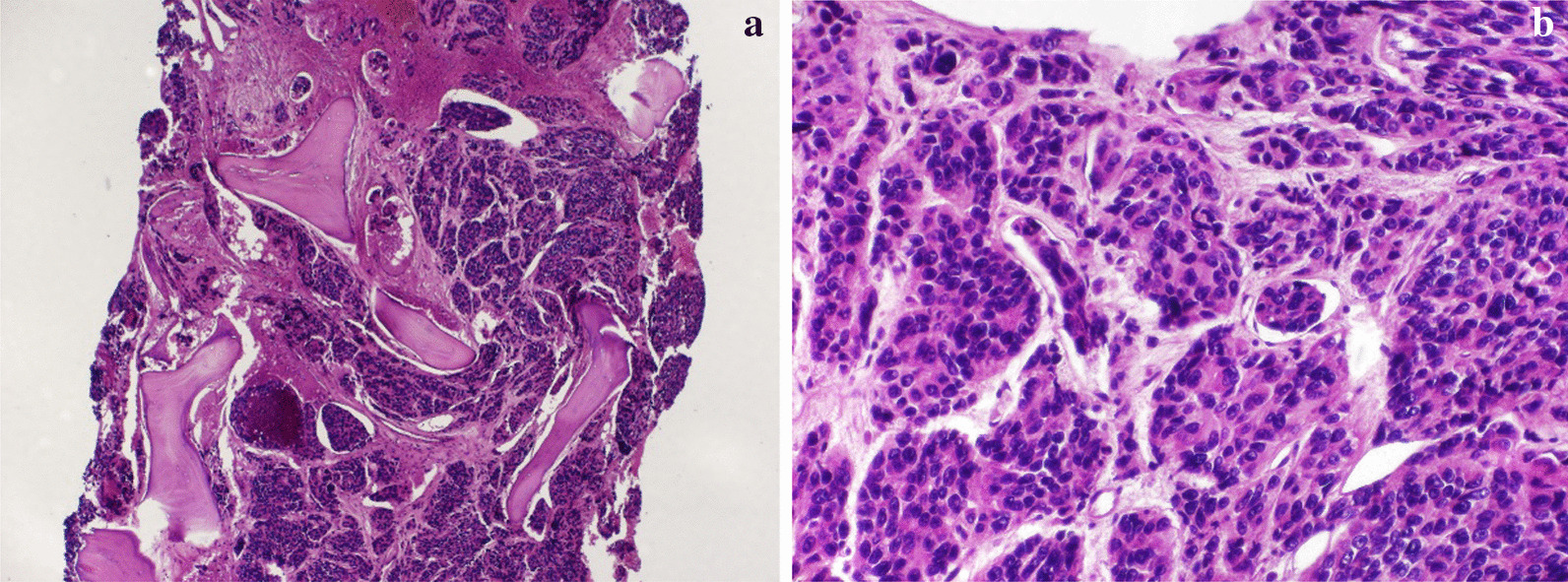
**a**, **b** Bone marrow biopsy demonstrating diffuse bone replacement by metastatic adenocarcinoma.** a** (×10),** b** (×20)

**Fig. 2:**
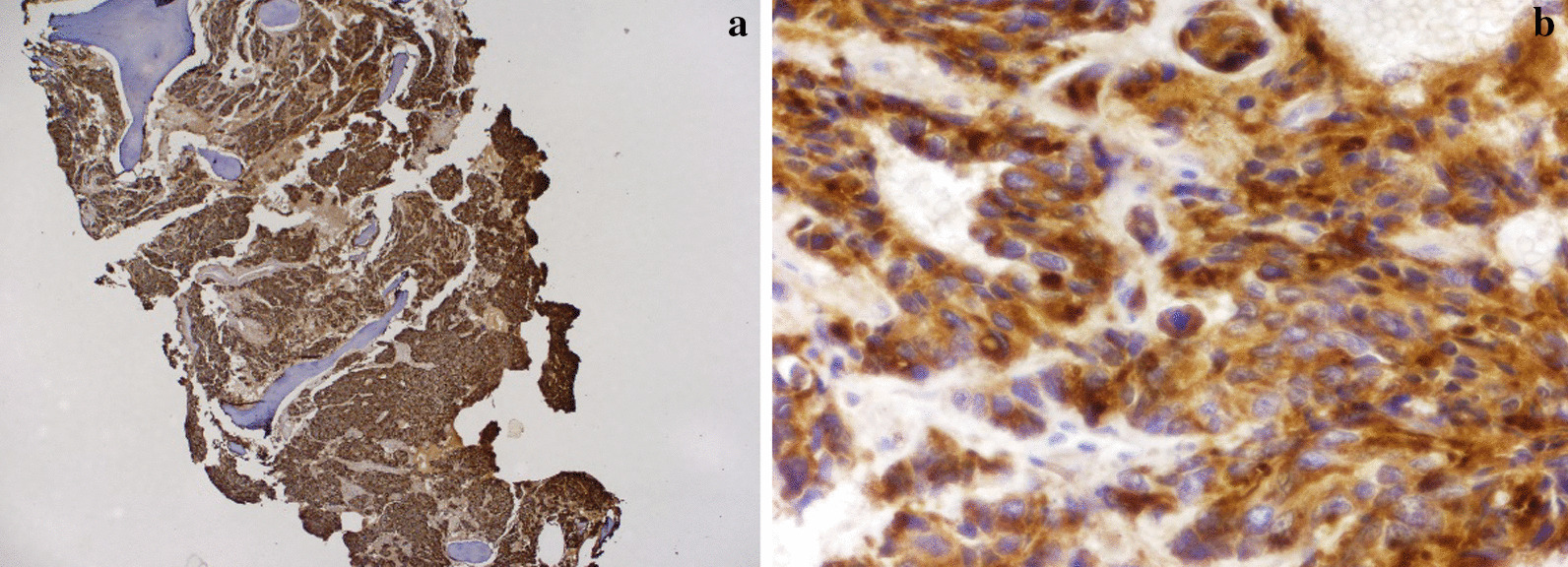
**a**, **b** Bone marrow replacement by prostate adenocarcinoma. Prostate-specific antigen stains were positive. ** a** (×10),** b** (×20)

To assess the etiology of the pancytopenia, bone marrow aspiration and biopsy were performed. Myelogram showed markedly increased cellularity in clumps, cell conglomerates with non-hematological characteristics, suggestive of spinal infiltration due to non-hematological neoplasia and the bone marrow biopsy showed diffuse bone marrow replacement by metastatic adenocarcinoma (Fig. 1). PSA stain was consistent with tumor cells of prostatic origin (Fig. 2).

Prostate examination revealed normal prostate, without nodularity and normal consistency. Because of this, another kidney US and abdomen CT were performed. US showed non-obstructive nephrolithiasis on the left and diffusely thickened walls of the bladder. CT of the abdomen showed areas of increased density, mainly in L4 vertebra posterior elements, in the context suggestive of secondary neoplastic involvement. Lymph node metastasis in the para-aortic, interaortocaval, and right iliac chains. The largest of them, near the confluence of the right common iliac vein with the inferior vena cava, measured 1.7 × 3.1 × 3.8 cm. Pulmonary nodules—the largest of them 1.0 cm in the middle lobe.

The disease staging was TXN1M1. He was evaluated by an oncologist and then started docetaxel and leuprolide. Additionally, zolendronic acid was started intravenously. He had no significant improvement of symptoms after four cycles and the oncology team decided to initiate palliative care. In the follow-up, the patient needed hemotransfusion support twice.

The patient is still alive 1 year after the presentation.

## Discussion

Prostate adenocarcinoma is the most frequent cancer type among men in Brazil, followed by skin cancer [2]. Prostate cancer is frequently asymptomatic, but the most frequent symptoms of prostate cancer are dysuria, increased urinary frequency, and urinary retention secondary to obstruction from prostate nodules.

This case is an interesting report for several reasons. 

First, the patient did not present any urinary symptoms. The patient did present other signs and symptoms secondary to metastasis involvement; in this case, bone marrow invasion causing pancytopenia.

Second, bone, lung, and liver are the most common metastatic sites for prostate cancer [12]. When bone metastases are present, in large quantity, bone marrow invasion could happen and cause pancytopenia. In this case, the patient had bone marrow invasion without large involvement of bones. The suppression of blood cell lines causing a pancytopenia is less common and generally a later finding in the disease course when compared to osteoblastic/osteoclastic activity [13].

Third, the patient had a normal prostate examination, and both US and CT did not reveal any prostate nodule. If a patient has increased PSA levels and an abnormal digital rectal examination, transrectal US is performed for diagnostic management [14]. Nevertheless, the detection of prostate cancer has limited diagnostic accuracy, with 40% sensitivity and 50% specificity for detecting prostate cancer on the basis of PSA levels [14]. For image diagnostics, magnetic resonance imaging (MRI) may have prognostic value in prostate cancer. A negative MRI had a negative predictive value of 84% in a large prospective study [15].

The prognosis of patients with prostate cancer with diffuse bone marrow involvement is very poor. A retrospective analysis of patients with metastatic prostate cancer, bone metastasis, and pancytopenia showed that the median survival was 3 weeks to 4 months [16].

Kunthur reported the successful treatment of a patient with castrate-resistant metastatic prostate cancer and severe pancytopenia with docetaxel chemotherapy [17]. In this case, our patient was transfusion dependent because of severe pancytopenia and there was a concern about docetaxel chemotherapy causing further worsening of cytopenia, but he still needed transfusion support and was unresponsive to docetaxel therapy.

## Conclusions

This report has scientific interest because of the pancytopenia as an initial manifestation of prostate cancer in a man without urinary symptoms and no prostate nodularity. The diagnosis was evaluated by bone marrow biopsy and positive PSA stain, and he then started treatment. On the basis of history and physical examination, complementary examinations were performed, supporting the diagnosis.

## Data Availability

The data and materials are available from the medical patients record at Clinical Hospital of State University of Campinas.

## References

[1] Ferlay J, Soerjomataram I, Ervik M, et al. GLOBOCAN 2012 v1.0, cancer incidence and mortality worldwide. Lyon: IARC, 2013. (IARC Cancer Base, 11). http://globocan.iarc.fr. Accessed 10 May 2019.

[2] INCA. Instituto Nacional de Câncer José Alencar Gomes da Silva. Coordenação de Prevenção e Vigilância. Estimativa 2018: incidência de câncer no Brasil / Instituto Nacional de Câncer José Alencar Gomes da Silva. Coordenação de Prevenção e Vigilância. – Rio de Janeiro: INCA, 2017.

[3] Carlsson S, Assel M, Ulmert D, et al. Screening for prostate cancer starting at age 50–54 years. A population-based cohort study. Eur Urol. 2017;71(1):46–52. 10.1016/j.eururo.2016.03.026 PMC5061609 27084245

[4] Albright F, Stephenson RA, Agarwal N, et al. Prostate cancer risk prediction based on complete prostate cancer family history. Prostate. 2015;75(4):390–8. 10.1002/pros.22925 PMC4293302 25408531

[5] Kamangar F, Dores GM, Anderson WF. Patterns of cancer incidence, mortality, and prevalence across five continents: defining priorities to reduce cancer disparities in different geographic regions of the world. J Clin Oncol. 2006;24(14):2137–50. 10.1200/JCO.2005.05.2308 16682732

[6] Vickers AJ, Ulmert D, Sjoberg DD, et al. Strategy for detection of prostate cancer based on relation between prostate specific antigen at age 40–55 and long term risk of metastasis: case-control study. BMJ. 2013;346:f2023. 10.1136/bmj.f2023 PMC3933251 23596126

[7] Carlsson S, Assel M, Sjoberg D, et al. Influence of blood prostate specific antigen levels at age 60 on benefits and harms of prostate cancer screening: population based cohort study. BMJ. 2014;348:g2296. 10.1136/bmj.g2296 PMC3968958 24682399

[8] Mottet N, Bellmunt J, Briers E, et al. EAU–ESTRO–ESUR–SIOG guidelines on prostate cancer. Presented at the EAU Annual Congress Copenhagen 2018. 978-94-92671-02-8. Arnhem: EAU Guidelines Office.

[9] National Institut of Health. Surveillance, Epidemiology, and End Results Program. Annual Report to the Nation on the Status of Cancer. https://seer.cancer.gov/statfacts/htlm/prost.htm. Accessed 8 Jan 2019.

[10] Denmeade SR, Isaacs JT. A history of prostate cancer treatment. Nat Rev Cancer. 2002;2(5):389–96. 10.1038/nrc801 PMC4124639 12044015

[11] Zhu ML, Horbinski CM, Garzotto M, et al. Tubulin-targeting chemotherapy impairs androgen receptor activity in prostate cancer. Cancer Res. 2010;70:7992–8002. 10.1158/0008-5472.CAN-10-0585 PMC2978028 20807808

[12] Bubendorf L, Schopfer A, Wagner U, et al. Metastatic patterns of prostate cancer: an autopsy study of 1,589 patients. Hum Pathol. 2000;31(5):578–83. 10.1053/hp.2000.6698 10836297

[13] Ahmad A, Tan W. Atypical presentation of prostate cancer and the workup of an adenocarcinoma of unknown primary. World J Oncol. 2012;3(4):187–90. 10.4021/wjon482w PMC5649842 29147303

[14] Chen FK, de Abreu ALC, Palmer SL. Utility of ultrasound in the diagnosis, treatment, and follow-up of prostate cancer: state of the art. J Nucl Med. 2016;57(Suppl 3):13S-18S. 10.2967/jnumed.116.177196 27694164

[15] Filson CP, Natarajan S, Margolis DJ, et al. Prostate cancer detection with magnetic resonance-ultrasound fusion biopsy: the role of systematic and targeted biopsies. Cancer. 2016;122(6):884–92. 10.1002/cncr.29874 PMC4777653 26749141

[16] Nieder C, Haukland E, Pawinski A, et al. Anaemia and thrombocytopenia in patients with prostate cancer and bone metastases. BMC Cancer. 2010;10:284. 10.1186/1471-2407-10-284 PMC2894796 20540802

[17] Kunthur A. A castrate-resistant metastatic prostate cancer patient with severe pancytopenia, successfully treated with docetaxel chemotherapy. J Oncol Pharm Pract. 2019;26:1254. 10.1177/1078155219890653 31775579

